# Rediscovery of Rhabdomastix (Rhabdomastix) incapax Starý, 2005 (Diptera, Limoniidae), a crane fly species flightless in both sexes and probably endemic to Sardinia

**DOI:** 10.3897/zookeys.498.9446

**Published:** 2015-04-21

**Authors:** Jaroslav Starý, Jindřich Roháček

**Affiliations:** 1 Neklanova 7, CZ-779 00 Olomouc-Nedvězí, Czech Republic; 2 Silesian Museum, Nádražní okruh 31, CZ-746 01 Opava, Czech Republic

**Keywords:** Diptera, Limoniidae, Rhabdomastix (Rhabdomastix) incapax, new female, crane fly, wing reduction, ecology, behaviour

## Abstract

Rediscovery of Rhabdomastix (Rhabdomastix) incapax Starý, 2005 in Sardinia made it possible to update the description of the male and to provide the first description of the female of this species. Notes on the wing reduction, ecology, and behaviour of this species are appended.

## Introduction

Rhabdomastix (Rhabdomastix) incapax Starý, 2005 was described from a single teneral male collected in Sardinia. This holotype is preserved in ethanol and deposited in the Zoologisches Forschungsmuseum Alexander Koenig, Bonn, Germany. The male holotype is peculiar in having very long antennae, nearly two thirds the length of the entire body, and reduced wings, seemingly incapable of flight, reaching to the posterior margin of abdominal segment 4 (Starý 2005, Figs 1–2). The type locality was verbatim given as “… Rio de s’Éleme / S.S.389 …“ (Starý 2005: 490). Whereas the record for the Éleme river was clear, it turned out only later that „SS389“ is the number of the road from Monti to Alà dei Sardi (and further to Núoro). Thus, the type locality of Rhabdomastix (Rhabdomastix) incapax could be inferred as being near the bridge over the Éleme river on road SS389 (Province Olbia-Tempio).

Sardinia was visited in May 2014, and since the locality in question was within reach by car from our residence, we included it in our collecting plan. A fairly numerous population of Rhabdomastix (Rhabdomastix) incapax was found at the type locality and also recorded this species on another site in north-east Sardinia. This made it possible to provide additional information on this peculiar species. We here give some additions to the description of the male, describe the previously unknown female, and append notes on wing reduction, ecology and behaviour of the species.

## Material and methods

**Figures 1–3.**
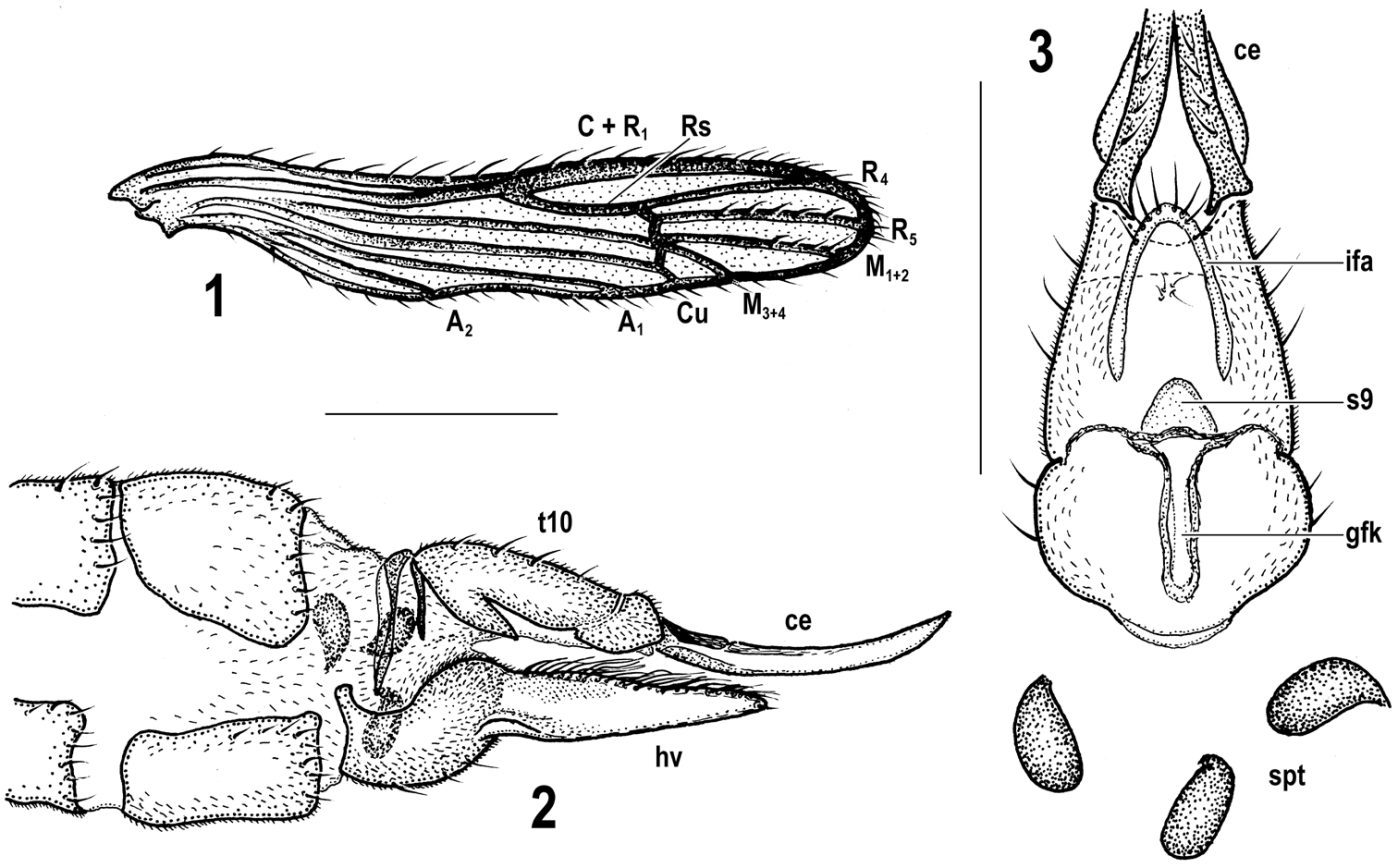
Rhabdomastix (Rhabdomastix) incapax. **1** Male wing **2–3** Female terminalia, general view, lateral (**2**) and internal structures, ventral (**3**). Scale bars 0.5 mm. ce – cercus; gfk – genital fork (vaginal apodeme); hv – hypogynial valve; ifa – infra-anal plate; spt – spermathecae; s9 – sternite 9; t10 – tergite 10.

The morphological terminology adopted here essentially follows McAlpine (1981). Designation of the wing veins is given in Fig. 1, and some special parts of the female terminalia of *Rhabdomastix* are referred to in Fig. 3 (cf. also figures in Starý 2003, 2004). Female terminalia were prepared by boiling in a solution of 10% KOH and preserved in glycerine in a small plastic tube pinned below the associated specimen. Line drawings were made using a drawing tube (*camera lucida*) attached to a compound microscope. Measurements were made using an ocular grid. Live specimens were photographed in special boxes by a digital camera (Canon EOS 60D) with a macro lens (Canon MP-E 65 mm 1–5×) and a ring macro flash (Canon MR-14EX).

## Results and discussion

### Redescription

#### Rhabdomastix (Rhabdomastix) incapax

Taxon classificationAnimaliaDipteraLimoniidae

Starý, 2005

**Figures 4–7.**
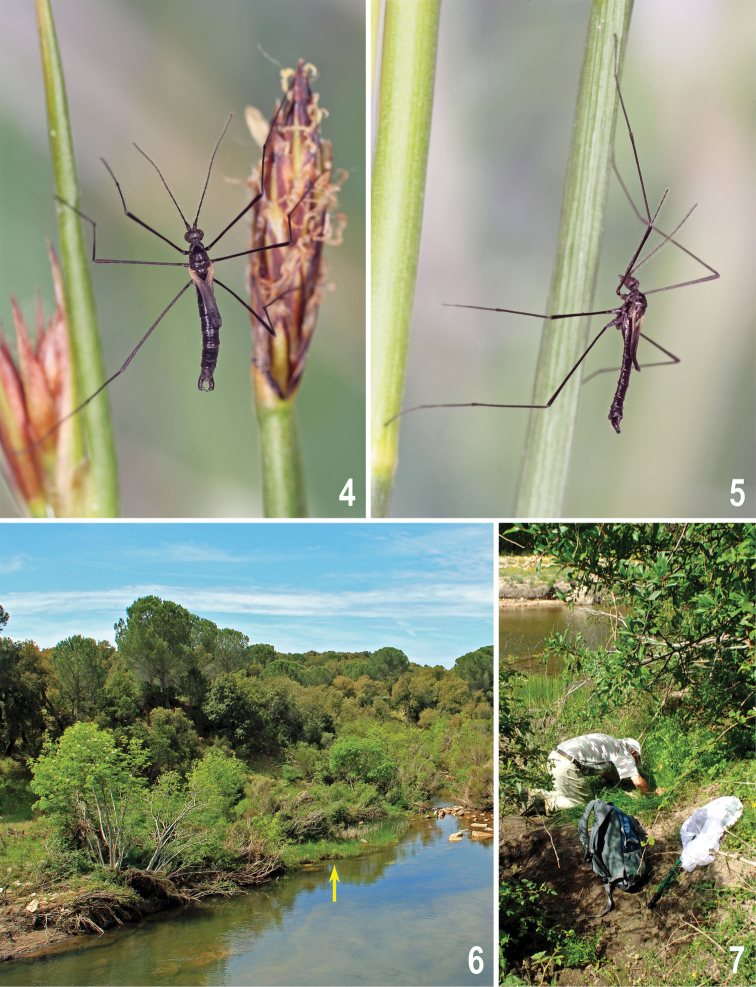
Rhabdomastix (Rhabdomastix) incapax, photographs of a live specimen, habitats, and collecting. **4–5** Rhabdomastix (Rhabdomastix) incapax, male on *Eleocharis* and *Juncus* stems and inflorescences **6** Éleme river taken from the bridge on road 389 (type locality, habitat of Rhabdomastix (Rhabdomastix) incapax is arrowed) **7** Collecting Rhabdomastix (Rhabdomastix) incapax in growth of *Eleocharis palustris* on the site arrowed in Fig. 6. Photographs by J. Roháček (4–6) and M. Vála (7).

**Figures 8–11.**
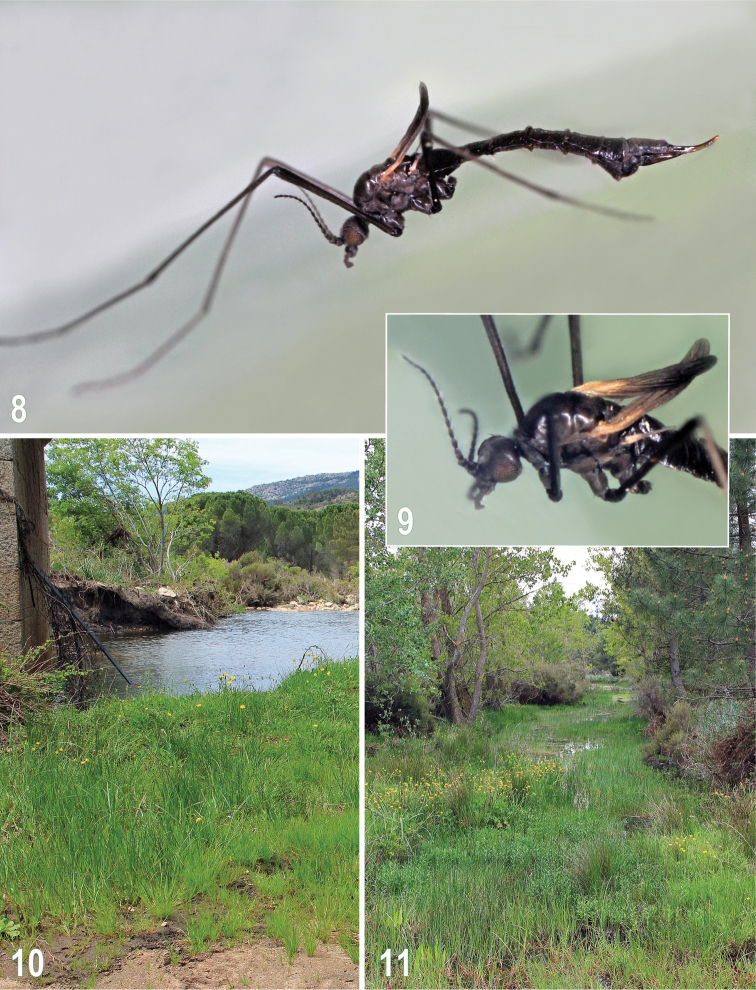
Rhabdomastix (Rhabdomastix) incapax, photographs of a live specimen and habitats. **8–9** Rhabdomastix (Rhabdomastix) incapax, female **10** Another habitat of Rhabdomastix (Rhabdomastix) incapax at the type locality (under the bridge, with *Juncus* sp. predominating) **11** Locality of Rhabdomastix (Rhabdomastix) incapax at Mazzinaiu nr. Alà dei Sardi, 6.6 km NE. Photographs by J. Roháček.

Figs 1–3 , 4–5 , 8–9

Rhabdomastix (Rhabdomastix) incapax Starý 2005: 490 (original description), Figs 1 (general view), 2 (male antenna), 3 (male palpus), 4 (male terminalia).

##### Description of male.

In general, the male was adequately described structurally in the original description. In contrast to the holotype, which was described as dirty yellow due to the teneral state and preservation in ethanol, the body of the fully-emerged specimen is shiny black throughout, with only bases of the wing rudiments and bases of the halteres light orange-yellow (Figs 4–5, 8–9). In dry-mounted material, the bases of the wings and halteres are darker and less conspicuous. Whereas the body, especially the abdomen, becomes somewhat wrinkled in dry-mounted specimens, in material preserved in ethanol an opposite process often occurs. This may change proportions and measurements from what was stated in the original description. The measurements of the holotype were given as follows: body length 4.0 mm, wing length 1.5 mm, antenna 2.5 mm (Starý 2005: 490). We here give the measurements based on dry-mounted males (see Material examined): body length 2.7–3.6 mm, wing length 1.4–1.8 mm, antenna 2.3–3.0 mm. For a better idea of live specimens, Figs 4–5, 8–9 should be consulted. Wing and wing venation of the male were partly described in the original description. The male wing is here illustrated (Fig. 1), with emphasis on the fact that veins R_5_ and M_1+2_ have a few macrotrichia dorsally and vein A_2_ is apparent. As described for the female below, the whole abdomen of the male is likewise densely covered with well-developed, spinoid microtrichia, not only segment 9 and the gonocoxite, as follows from the original description.

##### Description of female.

In general appearance resembling male. Body length 3.1–3.5 mm, wing length 0.9–1.3 mm, antenna 1.0–1.1 mm (dry-mounted specimens).

**Head.** Antenna 15-segmented, considerably shorter than that of male, reaching to base of halter (Figs 8–9), distinctly longer than antenna of most other European Rhabdomastix (Rhabdomastix) females [except for female Rhabdomastix (Rhabdomastix) hirticornis (Lackschewitz, 1940), subequal in length of antenna to female Rhabdomastix (Rhabdomastix) incapax]. Flagellomeres from ovoid to long-ovoid, gradually narrowed towards apex of antenna. Verticils on flagellomeres sparse, more or less decumbent, shorter than respective segments. Palpus short, as in male (cf. Starý 2005, Fig. 3).

**Thorax** slender as in male, with only a few short setae dorsally. Wing as in male (cf. Fig. 1) including venation, only slightly shorter and narrower (Figs 8–9), in general very dark to blackish, obscure yellow at base in dry-mounted specimens. Halter as in male, reaching to about half length of abdominal segment 2, obscure yellow at base, otherwise dark. Legs shorter than those of male, femora somewhat thickened distally, length ratio of male and female femur about 1.2: 1.

**Abdomen** slightly stouter than in male, with only rows of short setae along posterior, and partly lateral, margins of segments as in male, and densely covered with well-developed, spinoid microtrichia, both on sclerites and membranes, except for cercus and hypogynial valve. **Female terminalia** (Figs 2–3). Cercus slender, gently upturned, slightly longer than tergite 10. Hypogynial valve extending to about one third length of cercus. Internal structures much as in other European Rhabdomastix (Rhabdomastix) species (cf. Starý 2004), differing only in details. Infra-anal plate slender, strongly arched, more or less horseshoe-shaped, with several setae at posterior margin; sternite 9 membranous, little-distinct, generally triangular; genital fork (vaginal apodeme) comparatively slender and long, with darkened edges; spermathecae three, darkly pigmented, medium-sized, long-ovoid to reniform, somewhat narrowed in portion closer to duct, practically without sclerotized parts of ducts (Figs 2–3).

##### Material examined.

Italy: Sardinia (north-east): Monti, 8.1 km S, Rio de s’Éleme, road bridge (riverside vegetation), 465 m, 40°44'N, 9°22'E (Figs 6, 7, 10), 7.v.2014, 3 ♂, 1 ♀, 12.v.2014, 24 ♂, 2 ♀ (J. Roháček & J. Starý leg.); Mazzinaiu nr. Alà dei Sardi, 6.6 km NE, marshy vegetation along brook, 508 m, 40°45'N, 9°25'E (Fig. 11), 7.v.2014, 1 ♂ (M. Vála leg.) (all in coll. J. Starý, Olomouc, Czech Republic).

##### Discussion.

Noticeably, Rhabdomastix (Rhabdomastix) incapax is the only species among European *Rhabdomastix* with reduced wings. The shiny black body colouration is another distinguishing character evident at first sight. The female antennae of Rhabdomastix (Rhabdomastix) incapax are longer than those of females of the majority of other species. Rhabdomastix (Rhabdomastix) hirticornis is the single other European species that has conspicuously sexually dimorphic antennae, corresponding in length, relative to the size of the species, to those of Rhabdomastix (Rhabdomastix) incapax. Female terminalia of the latter species are of general structure usual for Rhabdomastix (Rhabdomastix), the most indicative character being the shape of the spermathecae, which are considerably elongate (Figs 2–3, for various details in other species, see figures in Starý 2004, 2005).

##### Distribution.

Italy: Sardinia. Considering the flightlessness of Rhabdomastix (Rhabdomastix) incapax and, consequently, its very limited dispersal abilities, the species is most probably endemic to the island.

### Wing reduction in Rhabdomastix (Rhabdomastix) incapax

Wing-reduced Diptera are often characterized by having other body extremities shortened as well, namely the antennae and legs. The antennae in the male of Rhabdomastix (Rhabdomastix) incapax, however, are very long, and the legs are considerably slender (Figs 4–5), corresponding to those of some fully-winged species of this genus. It is worthy of mention that, in Rhabdomastix (Rhabdomastix) incapax, reduction of the wings has not yet achieved a strongly brachypterous appearance, but rather seems to have advanced towards what could be termed a stenopterous condition. As to the wing length, the species in question shows a certain resemblance to some unrelated species of Limoniidae with shortened wings, probably flightless, such as females of Phylidorea (Phylidorea) heterogyna (Bergroth, 1913), or both sexes in Molophilus (Molophilus) ater (Meigen, 1804). In the latter two species, in contrast to Rhabdomastix (Rhabdomastix) incapax, the legs are correspondingly shorter and stouter. On the other hand, in Dicranomyia (Dicranomyia) reductissima (Alexander, 1952) [= *lindrothi* (Tjeder, 1963)] (Limoniidae), described from Tibet (as *lindrothi* from Alaska), a species micropterous in the male (female unknown), the legs are very slender and the antennae normally developed (Tjeder 1963). [Note: A form characterized as stenopterous in both sexes, interpreted as belonging to this species, was recorded from Mongolia and Tuva Region in Russia (Savchenko 1972).] Another example of long-legged brachypterous species may be Symplecta (Symplecta) holdgatei (Freeman, 1962) from the Gough Island in the Atlantic Ocean (Jones et al. 2003). This indicates that reduction of the wings and shortening of the antennae and legs may not necessarily be correlated and may be subject to different selective pressures.

Hackman (1964) proposed several categories of wing-reduced Diptera according to the climatic conditions of their habitats and modes of their lives. In addition to a few comparatively curious cases related to parasitism, unknown in tipulomorphans, wing reduction is mostly explained as adaptation to severe environmental conditions. These include, above all, low temperature inhibiting flying activity (see also Byers 1961, Mani 1962, Martinovský and Starý 1969) under arctic, alpine or nival conditions. For some insular species or those living on the seashore, strong winds may favour selection towards wing reduction as a factor generally hindering the flight, not explicitly preventing the insects from being blown out to sea, as sometimes suggested (cf. Huxley 1945 ex Hackman 1964). In Tipulidae, brachyptery of some females (*Tipula*, mostly subgenus *Vestiplex*) may be correlated with their deep-boring mode of oviposition, in relation to low temperature, or wind exposure, or both (Hemmingsen 1956).

Rhabdomastix (Rhabdomastix) incapax, however, occurs in the warm Mediterranean subregion, at moderate altitudes (465–508 m), and on inland sites sheltered from wind. Hence, wing reduction resulting from adaptation to the life in terricolous habitats, particularly among dense low vegetation, may be the case for this species (cf. Hackman 1964; as known in *Molophilus ater*). This could indicate that although the size of wings has limited dispersal ability, but the habitat closer to water might have helped the species to disperse by water carrying. Wing reduction in Rhabdomastix (Rhabdomastix) incapax may have been also supported by combination of high humidity and secretive life on sandy to muddy ground at bases of low graminoid plants, similar to what is observed for *Crumomyia pedestris* (Meigen, 1830) (Sphaeroceridae), inhabiting marshland habitats (Roháček 1975, 2012).

### Ecology and behaviour

All European *Rhabdomastix* species, in fact their larvae, require flowing water, and the adults live under riparian conditions, being closely associated with sandy or gravely banks of streams and larger rivers, overgrown with scattered, low vegetation. Even some fully-winged species may be observed sitting or crawling about on plants or on the ground, but are rarely seen flying (Starý 2003). A few specimens of Rhabdomastix (Rhabdomastix) incapax were at first discovered by us by sweeping a low riverbank vegetation, but the best technique to collect this species is by crawling on all fours, pulling apart tufts of plants and then aspirating specimens directly into the pooter (Fig. 7). The adults of Rhabdomastix (Rhabdomastix) incapax were found in the growths of the spikerush (*Eleocharis palustris*) and a small rush (*Juncus* sp.). Patches of these plant species occurred on sandy to muddy banks of the Éleme river (Figs 6, 10). Among this low vegetation, the adults, mostly males, significantly outnumbering females, were moving rather quickly, and when stimulated by our disturbance, they climbed up onto plant stems, making sometimes short jumps from these down on the wet ground.

Rhabdomastix (Rhabdomastix) incapax was collected not only at the Éleme river, but one specimen (see Material examined) was also swept from the vegetation on another site, approximately 6.8 km south-east of the type locality, with a somewhat different habitat. This included a slowly flowing brook (Fig. 11) with marshy littoral vegetation, containing, among other plants, low growths of a small *Juncus* sp. This indicates that the species may be more widely distributed in Sardinia, and not restricted only to banks of larger rivers.

## Supplementary Material

XML Treatment for Rhabdomastix (Rhabdomastix) incapax
